# An Examination of the Relationship Between Coaches’ Transformational Leadership and Athletes’ Personal and Group Characteristics in Elite Youth Soccer

**DOI:** 10.3389/fpsyg.2021.707669

**Published:** 2021-07-16

**Authors:** Martin K. Erikstad, Rune Høigaard, Jean Côté, Jennifer Turnnidge, Tommy Haugen

**Affiliations:** ^1^Department of Sport Science and Physical Education, University of Agder, Kristiansand, Norway; ^2^School of Kinesiology and Health Studies, Queen’s University, Kingston, ON, Canada

**Keywords:** transformational coaching, self-regulation, motivational climate, athlete satisfaction, group cohesion

## Abstract

There is a growing body of the literature highlighting the positive impact of transformational leadership behaviours across contexts, including sport. However, there is a lack of knowledge of this relationship within elite sport settings. Thus, the purpose of the present study was to examine the relationship between elite youth athletes’ perceptions of coaches’ transformational coaching-behaviours and variables that have been linked to transformational leadership in other settings (i.e., group cohesion, motivational climate, self-regulation of learning and athlete satisfaction). Norwegian elite youth soccer players (*n* = 753) selected into the national talent development program completed questionnaires to measure the variables of interest. Using structural equation modelling, results revealed a positive path from transformational leadership to both task and social cohesion, task-oriented motivational climates, self-regulation of learning and athlete satisfaction. Finally, a negative path from transformational leadership to ego-oriented climates was identified. The findings are in line with previous research in associating transformational leadership behaviours with adaptive outcomes, and further indicating that such relationships may also be valid in elite sport contexts.

## Introduction

Engagement in sport can lead to a variety of desirable outcomes for individual participants, which can be grouped into participation, personal development and performance (Côté et al., 2014). However, these outcomes do not occur because of engagement alone, and the coach is generally considered highly influential for an athlete’s experiences and development in sport (e.g., skill aquisition, motivation and satisfaction; Côté and Gilbert, 2009; Arthur et al., 2017). For instance, an investigation of Australian high-performance athletes from 34 sports revealed that coaches were perceived as critical and highly influential for their development throughout their career (Gulbin et al., 2010). Besides direct learning of sport skills, the coach can influence athletes and team outcomes through appropriate interactions and behaviours. Studies have highlighted the importance of coaches’ leadership behaviours on a diverse range of outcomes, such as group cohesion (Smith et al., 2013), motivational climate (Álvarez et al., 2019), athlete satisfaction (Nazarudin et al., 2009) and aspects related to self-regulation of learning (i.e., goal setting and initiative; Vella et al., 2013).

Broadly, the term leadership is used to describe the process whereby an individual influences a group of individuals to achieve a common goal (Northouse, 2018) and several definitions and models have been proposed to understand coach leadership in sport (e.g., Chelladurai, 1990; Horn, 2008; Côté and Gilbert, 2009; Burton et al., 2019). Côté and Gilbert (2009, p. 316), have proposed that effective coaching can be defined as “*the consistent application of integrated professional, interpersonal, and intrapersonal knowledge to improve athletes’ competence, confidence, connection and character in specific coaching contexts.*” Drawing upon this definition, Vella et al. (2010) suggested that coach leadership can be understood as a process of interpersonal influence, dependent upon the relationship between coach and athlete, that facilitates the athlete outcomes of competence, confidence, connection and character. As such, coach leadership provides a lens for understanding coaches’ interpersonal behaviours and how interpersonal behaviours impact athlete outcomes (Chelladurai, 1993). Thus, a commonality between the various definitions and models is that effective leadership in sport is dependent on multiple interacting factors, including coach and athlete characteristics and the situational context (Chelladurai, 1990; Horn, 2008; Côté and Gilbert, 2009). Furthermore, it is suggested that coaches’ behaviours are more likely to be consistent when aligned with their underlying values and beliefs (Becker, 2013).

Transformational leadership is one of the most researched and practical conceptual framework to understand the effect of different leadership behaviours on followers’ outcomes (Bass, 1985; Bass and Riggio, 2006; Barling, 2014). Specifically, transformational leaders inspire, challenge and motivate followers to reach their potential, and their leadership style is characterised by the four I’s; idealized influence (behave as role models), inspirational motivation (inspire and express confidence in followers potential), intellectual stimulation (encourage followers to develop independence and consider issues from different perspectives) and individual consideration (show care and interest in followers’ feelings and perspectives; Bass and Riggio, 2006). The concept of transformational leadership has also been incorporated into the Multidimensional Model of Sport Leadership (Chelladurai, 2007; Riemer, 2007), whereby successful coaches influence member and situational characteristics through the use of transformational leadership behaviours. Although transformational leadership has been investigated in various contexts (e.g., organisational psychology, healthcare and education; Turnnidge and Côté, 2016), there is an increased focus on transformational leadership in the sport setting (Arthur et al., 2017; Lawrason et al., 2019) and observational evidence has recently contextualised coaches’ transformational leadership behaviours within the sport setting (Turnnidge and Côté, 2019a).

Turnnidge and Côté (2016) concluded in their review of transformational leadership across a variety of disciplines that transformational leadership behaviours generally have been associated with positive outcomes, including how followers feel about themselves, their relationships, their environment and their tasks. Sport research supports the association between transformational coaching behaviour, and positive athlete outcomes and nurturing coach-athletes’ relationships. For example, transformational leadership has been positively associated with athletes’ wellbeing (Stenling and Tafvelin, 2014), prosocial behaviour (Tucker et al., 2010), satisfaction (Kao and Tsai, 2016), and enjoyment and perceived competence (Price and Weiss, 2013). In their study on 455 recreational adolescent soccer players, Vella et al. (2013) found perceptions of transformational coaching behaviours to be positively associated with personal and social skills, goal setting and initiative. Further, studies have investigated potential mediators in facilitating positive outcomes, as athletes’ need satisfaction can mediate the relationship between transformational leadership and wellbeing (Stenling and Tafvelin, 2014) and that intrinsic motivation can mediate the relationship between transformational leadership and sport performance (Charbonneau et al., 2006).

Research has also indicated that transformational leadership can foster group dynamics, such as cohesion (Callow et al., 2009; Smith et al., 2013) and task-oriented motivational climates (Álvarez et al., 2019). Although transformational coaching research in the sport context is relatively new, the studies appear to be consistent in indicating a positive association between transformational coaching and positive athlete outcomes. However, there are limitations on the present state of knowledge on transformational coaching in sport that limits our understanding.

While youth sport groups have been the primary focus of transformational coaching research in sport, Arthur et al. (2017) noted in their review that most of the studies have been conducted on less skilled athletes. As a recent exception, Subijana et al. (2021) investigated 223 elite youth athletes from a variety of sports and found positive associations between transformational leadership and intrinsic motivation. Further, Smith et al. (2017) conducted a qualitative study on transformational leadership behaviours in an elite sport environment. Through interviews of nine professional cricket athletes, the authors identified multiple transformational coaching behaviours to be present in elite sport, including high-performance expectations and individual consideration. Furthermore, the findings indicated that transformational coaching behaviours had positive effects on athletes (e.g., that high expectations by the coach could lead to increased effort). However, it should be noted that defining elite athletes are not straightforward, and that factors, such as competitiveness of the domain, and national and globally competitiveness of the sport, should be considered when classifying elite samples (Swann et al., 2015). Thus, it is worth mentioning that transformational leadership studies typically have examined “elite” athletes from less globally widespread/competitive sports, such as floorball (Stenling and Tafvelin, 2014) and ultimate frisbee (Smith et al., 2013).

Indeed, Evans et al. (2017) have pinpointed that differences in the sport structures (e.g., competition level) can substantially impact athletes sport experience. This notion is underpinned by findings from Australian athletes, where Gulbin et al. (2010) found that as competition level increased, athletes preferred coaching behaviours changed from more interpersonal and pedagogical qualities (e.g., ability to motivate and teach) to more technical qualities (e.g., detailed knowledge of the sport). Elite athletes’ preferred coach behaviours may thereby differ from less skilled athletes, and empirical findings from studies on less skilled athletes may not necessarily be transferred to elite athletes. For instance, some transformational leadership behaviours identified by Turnnidge and Côté (2019a) may be perceived as less important for performance in elite sport (e.g., discussing and modelling prosocial behaviours), whereas other behaviours may be perceived by coaches as having a more direct link to performance (e.g., providing challenging and meaningful tasks and roles). For athletes aiming to develop or demonstrate high-performance levels, important factors have been identified. Of relevance for the present work, studies have indicated a beneficial role of adaptive group processes, such as cohesion (i.e., a group’s tendency to stick together and remain united; Carron et al., 1998) and task-oriented motivational climates (e.g., focusing on effort and self-referenced comparisons) for athletes at both elite and non-elite levels (see, e.g., Martin et al., 2014; Harwood et al., 2015; Erikstad et al., 2018b). Furthermore, studies have shown that self-regulation of learning, which refers to athletes being proactive in their own learning process (Zimmerman, 1989), is associated with athletes’ capacity to train more effectively (Toering et al., 2009), and higher levels of performance (Erikstad et al., 2018a). Finally, dimensions related to thriving and wellbeing are considered prerequisites for athletic development and performance (Brown et al., 2018), thus indicating the important role of athlete satisfaction (see also Riemer and Chelladurai, 1998). While transformational leadership has been linked to both cohesion (Callow et al., 2009; Smith et al., 2013), task-oriented motivational climates (Álvarez et al., 2019) aspects of self-regulation of learning (e.g., goal setting and initiative; Vella et al., 2013) and satisfaction (Kao and Tsai, 2016), knowledge about these relationships among elite youth athletes seems scarce, particularly in highly competitive sports.

Given the lack of knowledge on outcomes associated with transformational coaching in elite youth sport settings, the purpose of the present study was to investigate the relationship between elite youth soccer athletes’ perceptions of coaches’ transformational leadership behaviours and group cohesion, motivation climate, self-regulation of learning and athlete satisfaction.

## Materials and Methods

### Participants

The sample consisted of Norwegian male elite youth soccer players (*n* = 753) that were selected for regional competitions at U14- (*n* = 363) or U13-level (*n* = 390). A regional team is associated with the Norwegian national talent development program and consist of approximately 25 of the most promising players from each age cohort between ages 13 and 16 within a soccer region. Players associated with the program received high-quality training and matches that were supplementary to their involvement at club level. While the frequency of these practice sessions varied due to several factors (e.g., geographical variation), it was approximately one session weekly. The sample thereby represents the most promising players in Norway within their respective age categories. Data from two regions were not collected, thus 16 of 18 soccer regions in Norway were represented in the present study. The present study was a part of a larger project that was conducted between 2015 and 2018 for which articles have been published (see Erikstad et al., 2018a,b).

### Measures

#### Transformational Leadership

Perceptions of transformational coaching behaviours was measured using the Global Transformational Leadership scale (GTL; Carless et al., 2000). Based on a summary of the literature on transformational leadership (i.e., Bass, 1985; Podsakoff et al., 1990), Carless et al. (2000) developed seven items that constituted a global construct of transformational leadership. Although the questionnaire originally aimed to measure transformational leadership perceptions in organisational settings, the questionnaire has previously been used in the sport context (Tucker et al., 2010). In line with Tucker et al. (2010), the questionnaire was adapted to fit the sport context (i.e., “staff” was replaced by “players” and “my leader” was replaced by “my coach”). Example of an item is: “My coach communicates a clear and positive vision of the future”. The items were rated on a 5-point Likert scale ranging from 1 (*rarely or never*) to 5 (*often, if not always*). Previous studies have indicated an acceptable internal consistency of the measure in both sport (Tucker et al., 2010) and non-sport (Nielsen and Munir, 2009) settings.

#### Group Cohesion

To assess group cohesion, the participants completed the Norwegian version of the YSEQ (Eys et al., 2009) that assesses task (eight items) and social cohesion (eight items). Item examples are “We like the way we work together as a team” (task cohesion), and “Some of my best friends are on this team” (social cohesion). Participants responded to each of the 16 items on a 9-point Likert scale anchored at 1 (*strongly disagree*) and 9 (*strongly agree*), with a higher score reflecting stronger perceptions of cohesion.

#### Motivational Climate

Perceptions of motivational climate was assessed using the Perceived Motivational Climate in Sport Questionnaire (PMCSQ; Selfriz et al., 1992). The instrument consists of 21 items to measure the performance climate (12 items) and mastery climate (nine items) within a sport group. The original instrument was developed within the basketball context and included stems for each question related to basketball, such as “On this basketball team…”. For this study, the stems were adjusted to “On this team…” to fit the soccer context. Example questions include “doing better than others is important” (performance climate), and “players try to learn new skills” (mastery climate). The responses were scored on a 5-point Likert scale. While the original instrument is scored from 1 (*strongly agree*) to 5 (*strongly disagree*), the scoring was reversed to be in accordance with other scales used in the present study (i.e., from strongly disagree to strongly agree). The present study used a Norwegian version of the instrument (Roberts and Ommundsen, 1996).

#### Self-Regulation of Learning

Self-regulation of learning was measured using a short Norwegian version of a football-specific self-regulated learning questionnaire (SRL; Toering et al., 2013). While the original instrument contains 22 items related to planning, evaluation and reflection, the short measure consists of eight items that represent a global measure of self-regulation. Example items are “During each practice session I check what I still have to do to reach my practice goal” and “After each practice session I think back and evaluate whether I did the right things to reach my practice goal.” Items were rated on a 5-point Likert scale ranging from 1 (*never*) to 5 (*always*). The short version of the instrument has previously been applied in Norwegian youth soccer setting (Erikstad et al., 2018a). The measure was also used in Erikstad et al. (2018b) in relation to cohesion.

#### Athlete Satisfaction

Athlete satisfaction was measured using a subscale from athlete satisfaction questionnaire (ASQ; Riemer and Chellandurai, 1998). ASQ is a 15 subscale instrument consisting of 56 items to measure various dimensions of athlete satisfaction (e.g., satisfaction with team performance, team social contribution and academic support). For the present study, the subscale related to “ability utilization” was used, consisting of five items that measure athletes’ satisfaction with how the coach uses and/or maximizes the individual athletes’ talents and abilities (e.g., “I was satisfied with the extent to which my role matches my potential”). Items were rated on a 7-point Likert scale, ranging from 1 (*not at all satisfied*) to 7 (*extremely satisfied*), with the midpoint (4) described as “moderately satisfied”.

### Procedures

The present study was approved by the ethical committee of the first author’s University and the Norwegian Social Science Data Services. Prior to data collection, the Football Association of Norway [Norges Fotballforbund (NFF)] was contacted to help recruit athletes for the project, and all regions and regional players were subsequently encouraged to participate. All regions (*n* = 18) in Norway were contacted by the researchers by email or telephone with an invitation to participate in the study with athletes selected for their male regional U-13 and U-14 teams. For participating regions (*n* = 16), questionnaires, information letter and a test protocol were distributed to a contact person in NFF, and an information letter was distributed to selected players and their parents. The players completed the questionnaires individually prior to a practice session before the season started under the supervision of a test leader in a classroom setting. The players were informed in writing and verbally that the survey was anonymous and voluntary, and that all information would be treated confidentially. For questions related to their coach, the players were asked to think of the coach they most frequently interacted with (i.e., their club coach). Completed questionnaires were collected and sealed into an envelope by the test leader, and further distributed to the first author by mail.

### Statistical Analysis

All analyses were performed with the Mplus 8.4 (Muthén and Muthén, 1998–2017) software. Independent Clusters Model Confirmatory Factor Analyses were performed to assess the psychometric properties of the questionnaires, and the hypothesised research model using transformational leadership to predict cohesion, motivational climate, athlete satisfaction and self-regulation was examined using structural equation modelling. The structural model contained transformational coaching as exogenous variable, and self-regulation of learning, athlete satisfaction, motivational climate and group cohesion as endogenous variables. Robust full information maximum likelihood estimator, which provides standard errors and a chi-square test statistic that are robust to non-normality was conducted (Satorra and Bentler, 1994).

Satorra-Bentler’s scale for chi-square difference tests was used to assess the overall fit of each measurement model. While non-significant SB *χ*^2^ values typically indicate a satisfactory fit, these values are sensitive to sample size (Hu and Bentler, 1999). Thus, the comparative fit index (CFI), Tucker-Lewis index (TLI), root mean square error of approximation (RMSEA) and the standardised root mean square residual (SRMR) were also inspected. In line with existing recommendations (Bentler, 1995; Hu and Bentler, 1999), acceptable fit was indicated with values of >0.90 for CFI and TLI, and values of ≤0.08 for RMSEA and SRMR (Marsh, 2007). Associations and extents of the estimates of factor loadings, intercepts, variances, residual variances and *z*-scores were inspected. Byrne’s (2012) recommendations were used to outline potential re-specifications of the measurement models.

## Results

Initial analysis of the factor structure of the GTL indicated an acceptable model fit [S-B *χ*^2^(*df* = 51, *n* = 754) = 73.57, *p* < 0.001; TLI = 0.939; CFI = 0.960; RMSEA = 0.076, 90% CI = 0.059–0.094; and SRMR = 0.031]. Thus, the initial GTL model was retained.

The initial model fit for the cohesion scale (YSEQ) was deemed not acceptable due to low TLI and high RMSEA values [S-B *χ*^2^(*df* = 24, *n* = 748) = 519.86, *p* < 0.001; TLI = 0.89; CFI = 0.90; RMSEA = 0.08, 90% CI = 0.07–0.08; and SRMR = 0.05]. In line with Byrne’s (2012) recommendations, and based on an inspection of the items, their relations and their theoretical similarities, two items pertaining to social cohesion were allowed to co-vary in a new model [i.e., “We contact each other often (phone, text messages and Internet)” co-varied with “I contact my teammates often (phone, text messages and Internet)]”. This resulted in an acceptable fit of the YSEQ model [S-B *χ*^2^(*df* = 102, *n* = 702) = 405.141, *p* < 0.001; TLI = 0.918; CFI = 0.930; RMSEA = 0.065, 90% CI = 0.058–0.072; and SRMR = 0.051].

Regarding motivational climate (PMCSQ), the results indicated a poor model fit [S-B *χ*^2^(*df* = 169, *n* = 748) = 760.883, *p* < 0.001; TLI = 0.780; CFI = 0.804; RMSEA = 0.070, 90% CI = 0.065–0.075; and SRMR = 0.065]. Thus, with the conceptual importance of motivational climate in the broader literature in mind, it was decided to create a short version of the instrument. Ommundsen et al. (2013) previously created a short version of the instrument based on factor loadings from the original Norwegian version (Roberts and Ommundsen, 1996). However, as these items were not identified in the two abovementioned studies, we decided to use the factor loadings from our initial model to create a short version with four items from each of the two subscales (ego climate: item number 4, 5, 6 and 7; task climate: item number 13, 15, 16 and 17). This resulted in an acceptable model fit for the abbreviated version of the PMCSQ [S-B *χ*^2^(*df* = 19, *n* = 748) = 41.531, *p* < 0.001; TLI = 0.969; CFI = 0.979; RMSEA = 0.051, 90% CI = 0.024–0.058; and SRMR = 0.033].

For SRL, the initial factor solution indicated non-acceptable fit indices. Therefore, a modified model was estimated where the residuals of two items were allowed to correlate (i.e., “After each practice session I think back and evaluate whether I did the right things to reach my practice goal” and “Each practice session I think back and evaluate whether I did the right things to become a better player”). Due to the similarities in wording and content for the two items (Byrne, 2012), this modification of the measurement model was perceived to be conceptually meaningful. The same modification was done in a recent article by Erikstad et al. (2018b). The abovementioned re-estimation resulted in an acceptable model fit [S-B *χ*^2^(*df* = 19, *n* = 748) = 64.899, *p* < 0.001; TLI = 0.953; CFI = 0.968; RMSEA = 0.057, 90% CI = 0.042–0.072; and SRMR = 0.031]. Results for the factor structure of ASQ indicated an acceptable model fit [S-B *χ*^2^(*df* = 5, *n* = 720) = 26.131, *p* < 0.001; TLI = 0.954; CFI = 0.977; RMSEA = 0.077, 90% CI = 0.049–0.107; and SRMR = 0.025].

The results of the structural mode indicated an acceptable fit [S-B *χ*^2^(*df* = 879, *n* = 757) = 1740,547, *p* < 0.001; TLI = 0.926; CFI = 0.931; RMSEA = 0.036, 90% CI = 0.034–0.038; and SRMR = 0.045]. The model (see Table 1) revealed a positive path from GTLS to SRL (*β* = 0.324, *p* < 0.001), athlete satisfaction (*β* = 0.511, *p* < 0.001), task-climate (*β* = 0.704, *p* < 0.001), social cohesion (*β* = 0.340, *p* < 0.001) and task cohesion (*β* = 0.616, *p* < 0.001). Finally, a negative path from GTL to ego climate (*β* = −0.430, *p* < 0.001) was identified. The correlations between the endogenous variables are presented in Table 1.

**Table 1:** Multivariate correlations and 95% CI.

	SRL	AS	EC	MC	SC	TC
**GTL**	0.324^**^ **(0.237, 0.410)**	0.511^**^ **(0.432, 0.589)**	−0.430^**^ **(−0.514, −0.346)**	0.704^**^ **(0.616, 0.792)**	0.340^**^ **(0.258, 0.423)**	0.616^**^ **(0.540, 0.692)**
SRL	–	0.126^*^ (0.019, 0.233)	0.154^**^ (0.051, 0.256)	0.062 (−0.064, 0.188)	0.047 (−0.046, 0.140)	0.043 (−0.058, 0.145)
AS		–	−0.150^**^ (−0.244, −0.056)	−109 (−0.036, 0.254)	0.193^**^ (0.093, 0.293)	0.316^**^ (0.218, 0.414)
EC			–	0.017 (−0.103, 0.136)	0.075 (−0.024, 0.173)	−0.012 (−0.112, 0.089)
MC				–	0.102 (−0.012, 0.216)	0.329^**^ (0.179, 0.479)
SC					–	0.558^**^ (0.454, 0.662)

Bold values are predictor on endogenous variables. GTL, global transformational leadership; SRL, self-regulation of learning; AS, athlete satisfaction; EC, ego climate; MC, mastery/task climate; SC, social cohesion; and TC, task cohesion.

* *p* < 0.05;

** *p* < 0.01.

## Discussion

The present study aimed to perform an examination of the relationship between elite youth athletes’ perceptions of coaches’ transformational coaching behaviours and group cohesion, motivational climate, self-regulation of learning and athlete satisfaction. Overall, the structural model demonstrated positive paths between transformational coaching and the adaptive outcomes investigated (i.e., both task and social cohesion, task climates, self-regulation of learning and athlete satisfaction). A negative path from transformational coaching to ego climates emerged. Generally, the findings are in accordance with transformational leadership theory (Bass and Riggio, 2006) and findings from previous research (see, e.g., Turnnidge and Côté, 2016; Arthur et al., 2017). The study also contributes to understanding the role of transformational leadership in more competitive contexts, which was highlighted as a limitation in the existing transformational coaching literature (Arthur et al., 2017).

For the group dynamics variables, the present study identified positive paths from transformational coaching to both task and social cohesion and mastery climates, and a negative path to ego climates. Regarding the relationship between transformational coaching and cohesion, two previous studies conducted on ultimate frisbee players have demonstrated similar findings (Callow et al., 2009; Smith et al., 2013). Specifically, by focusing on task cohesion, Callow et al. (2009) found that transformational leadership behaviours (e.g., fostering acceptance of group goals, promoting teamwork, high-performance expectation and individual consideration) positively predicted task cohesion. Smith et al. (2013) found similar results regarding task cohesion, while also finding that fostering acceptance of group goals and promoting teamwork predicted social cohesion. While no previous studies have, to our knowledge, investigated the relationship between transformational coaching and ego climates, the positive path between transformational coaching and task-oriented motivational climates is in line with previous research from the youth soccer context (Álvarez et al., 2019). However, it is worth mentioning that motivational climates and coaching behaviours may change as a function of athletes age, as competitive pressure often increases as athletes gets older (Skille, 2011). Thus, the association between transformational leadership and motivational climate among older elite athletes remains uncertain.

While transformational leadership serves as a broad framework for understanding coach leadership and athlete outcomes, it was previously noted that transformational leadership shares conceptual similarities with both autonomy-supported and mastery-oriented coaching (see Turnnidge and Côté, 2019b). For instance, providing athletes with choices and listen to their input are examples of autonomy supportive coaching and are closely related to transformational leadership behaviours, such as eliciting athlete input and showing interest in athletes’ needs. Previous research has demonstrated the link between autonomy-supportive coaching and task climates (Jõesaar et al., 2012). Similar, mastery-oriented coaching is characterised by focus on skill improvement, effort and cooperation and is closely related to transformational leadership behaviours of such as emphasizing the learning process and promoting collaboration (Bass and Riggio, 2006; Turnnidge and Côté, 2019a). Mastery-oriented coaching can lead to motivational climates that are more mastery-oriented and less ego oriented (Smoll et al., 2007), which is in accordance with the present findings. While both cohesion and task climates have generally been associated with adaptive outcomes (Martin et al., 2014; Erikstad et al., 2018b), ego climates have been associated with more maladaptive outcomes (see Harwood et al., 2015).

Further, the present study found a positive relationship between transformational leadership and self-regulation of learning. Self-regulation of learning is highlighted as a key component of deliberate practice (Tedesqui and Young, 2015) which appears beneficial for youth elite athletes that aim to optimize their sport development (McCardle et al., 2019). While less is knows about how coaches may promote self-regulation of learning, findings from non-elite contexts have associated transformational leadership with aspects of self-regulation of learning, such as goal setting and initiative (Vella et al., 2013). Indeed, intellectual stimulation is one of four key elements of transformational leadership and relates to behaviours that stimulate followers’ intellectual curiosity and innovative approaches (Bass and Riggio, 2006). Transformational leadership behaviours, such as emphasizing the learning process and sharing leadership responsibilities, may thereby be beneficial for developing self-regulatory skills (Turnnidge and Côté, 2019a).

Finally, the present study identified a positive path between transformational coaching and athletes’ satisfaction. Such findings may not be surprising, as transformational leadership behaviours in sport highlight the value of different roles and showing appreciation for athlete efforts (Turnnidge and Côté, 2019a). The findings can also be considered in line with previous empirical works, as studies have found positive associations between transformational leadership and elite youth athletes’ intrinsic motivation (Subijana et al., 2021), and that transformational leaders generally tend to have satisfied followers across contexts (Judge and Piccolo, 2004; Kao and Tsai, 2016).

At a broader level, the present study adds to the literature by indicating positive outcomes associated with transformational leadership in elite youth sport (Turnnidge and Côté, 2016; Arthur et al., 2017). Specifically, such relationships may be dependent on competitive levels investigated (Evans et al., 2017), and Arthur et al. (2017) have highlighted the need for empirical investigations of elite athletes to advance our understanding of transformational coaching. While some studies have been conducted on competitive athletes (Stenling and Tafvelin, 2014; Kao and Tsai, 2016; Smith et al., 2017), such studies have typically been focusing on smaller samples, university level and/or less competitive sports (see, e.g., Swann et al., 2015 for issues related to defining elite athletes). Although more research on elite and professional athletes is warranted, the present study extends current knowledge by using a sample of youth athletes at the highest national level in soccer.

The limitations of this study, however, need to be considered. Perhaps, the most significant limitations were the use of a global measure of transformational leadership which limits our understanding of the subcomponents of transformational coaching (i.e., idealized influence, inspirational motivation, intellectual stimulation and individual consideration). However, one should bear in mind that all existing measures of transformational leadership in sport have limitations (see, e.g., Arthur et al., 2017; Turnnidge and Côté, 2019a), and that the concise nature of this global measure allows us to collect data on a large sample of young elite athletes. Furthermore, the cross-sectional nature of the work does not allow establishing causal connections. Finally, as the sample consisted of a homogeneous group of youth soccer players selected at national level, the transferability to individual sports, female athletes or professional sports is uncertain.

In summary, the present study adds to the literature that has associated transformational leadership behaviours with adaptive athletes’ outcomes (e.g., Smith et al., 2013; Vella et al., 2013), and further indicates that such relationships may also be valid in youth elite contexts. As a practical recommendation, coaches could aim to increase their use of transformational coaching behaviours to increase the likelihood of athletes’ positive sport experiences. Notably, Turnnidge and Côté (2019a) have increased our understanding of such transformational coaching behaviours through their work including systematic observations. There are also indications that transformational coaching behaviours can be developed through evidence-informed coach development programs (see Lawrason et al., 2019), although more research is warranted. Moving forward, there is also a need for a sport-specific questionnaire that focuses on the behaviours of coaches that are known to influence athletes positively. One possibility for future research would be to use the observational research by Turnnidge and Côté (2019a) as a foundation for the development of a multidimensional sport-specific measure of transformational leadership.

## Data Availability Statement

The raw data supporting the conclusions of this article will be made available by the authors upon request, without undue reservation.

## Ethics Statement

The studies involving human participants were reviewed and approved by the Norwegian Centre for Research Data. Written informed consent to participate in this study was provided by the participants’ legal guardian/next of kin.

## Author Contributions

ME was the project leader. ME, RH, JC, JT, and TH contributed in developing the study and wrote and revised the manuscript. ME, RH, and TH collected and analysed the data. All authors contributed to the article and approved the submitted version.

### Conflict of Interest

The authors declare that the research was conducted in the absence of any commercial or financial relationships that could be construed as a potential conflict of interest.
